# Satellite-based estimates of groundwater depletion in the Badain Jaran Desert, China

**DOI:** 10.1038/srep08960

**Published:** 2015-03-11

**Authors:** Jiu Jimmy Jiao, Xiaotao Zhang, Xusheng Wang

**Affiliations:** 1 Department of Earth Sciences, The University of Hong Kong, Hong Kong, China; 2 School of Water Resources & Environment, China University of Geosciences, Beijing, China

## Abstract

Despite prevailing dry conditions, groundwater-fed lakes are found among the earth's tallest sand dunes in the Badain Jaran Desert, China. Indirect evidence suggests that some lakes are shrinking. However, relatively few studies have been carried out to assess the regional groundwater conditions and the fate of the lakes due to the remoteness and severity of the desert environment. Here we use satellite information to demonstrate an ongoing slow decrease in both lake level and groundwater storage. Specifically, we use Ice, Cloud, and land Elevation Satellite altimetry data to quantify water levels of the lakes and show overall decreases from 2003 to 2009. We also use water storage changes from the Gravity Recovery and Climate Experiment and simulated soil and water changes from the Global Land Data Assimilation System to demonstrate long-term groundwater depletion in the desert. Rainfall increase driven by climate change has increased soil water and groundwater storage to a certain degree but not enough to compensate for the long-term decline. If countermeasures are not taken to control the pumping, many lakes will continue to shrink, causing an ecological and environmental disaster in the fragile desert oases.

The Badain Jaran Desert (BJD), the core part of the only UNESCO Desert Geopark, is located in the western part of Inner Mongolia, China. The desert is the fourth largest in the world, covering an area of ~49,000 km^2^. It is bounded on the southeast by the Yabrai Mountains, which separate it from the Tengger Desert (Fig. 1a), and on the south and southwest by the Heli and Beidai Mountains, which separate it from the Hexi Corridor^1^. The elevation gradually decreases from about 1800 m in the southeast to about 1000 m in the northwest. The Heihe River on the west is the only runoff near the desert. A prominent feature of the BJD is mega sand dunes that average 330 m in height; some are the world's highest at up to 480 m.

The climate is typical of an extreme continental desert^2^. The annual mean temperature ranges from 9.5 to 10.3°C, with the lowest temperature of −10°C in January and the highest of 25°C in July. The rainy season is from June through August. Precipitation decreases from the southeast to the northwest, with a mean annual precipitation of less than 90 mm. Potential evaporation increases from the south to the north, with an annual average of more than 2500 mm^2^. Monthly changes in rainfall and temperature from the Alxa Youqi meteorological station, the nearest town to the desert, are shown in Supplementary Fig. S1. Although the rainfall inside the desert is much less than from this station, it is believed that the temporal rainfall fluctuation is similar. The annual snow in winter averages about 5.4 mm equivalent water height; the snow impacts soil water in the winter but overall snow is negligible compared to rainfall.

The BJD is characterized by numerous lakes among stationary sand dunes. Most lakes are located in the southeast. Currently, 72 lakes comprise a total water surface area of 23 km^2^ 3. The largest has an area of 1.5 km^2^ and a water depth of up of 15 m. The large lakes are perennial but small lakes, especially those with areas less than 0.2 km^2^, can be intermittent and extremely sensitive to climate and groundwater level. Except for a few freshwater lakes near the southeast margin of the desert, most of the lakes are saline (salinity up to 330 g/L)^1^.

The desert remained largely unstudied until the 1990s^2^. A detailed review on various geological, geomorphological and groundwater studies can be found in Dong et al (2013). Groundwater studies have been active mainly in recent years and no comprehensive regional hydrogeological studies have been conducted. So far, groundwater studies by the increasingly involved Chinese and overseas academic community have focused on sources of water to the lakes^2^,^4^,^5^. Possible water sources include direct precipitation in the watershed among the mega dunes or slope areas of the nearby mountains to the south^1^,^6^; paleo-source recharge, with the groundwater originating from precipitation that fell in the distant past^4^,^5^,^7^; and remote sources^8^,^9^,^10^. On the basis of similarities between the δ^18^O and δD composition of groundwater in the desert and in the mountainous areas^9^, a deep and large fault was speculated to exist between the Qinghai-Tibetan Plateau and Yabrai Mountains that directly conveys a huge amount of water to the desert^10^.

Both researchers and local herdsmen report field evidence indicating gradual shrinking of some lakes and falling lake levels^6^. Based on the distance from the shore of Badain Lake (Fig. 1b) to some poplars planted by local herdsmen, an estimated 10% of this lake's surface dried up between 1983 and 1999^6^. Regional groundwater flow direction estimated from lake water levels can provide information about the recharge relationship between the desert and neighboring areas. Such measurements have been taken using handheld GPS^11^; however, they cannot provide reliable information on water level and groundwater flow direction due to low vertical accuracy. So far, the only temporal water level measurements available are for lakes L5 and L6 from April 2010 to October 2011; these data indicate yearly water level fluctuations of about 25 cm^11^. This annual fluctuation is believed to be typical, as local herdsman observed that seasonal changes of the level in large lakes are small^6^.

In this study, we accurately estimate lake water level by analyzing satellite altimetry data and calculate the terrestrial water storage (TWS) change in the desert using satellite gravity data. Groundwater storage is then isolated by subtracting TWS from simulated soil and water changes obtained to study seasonal and long-term variations. The estimated water level and groundwater storage, together with the meteorological data, are examined with respect to the recharge source of the groundwater and implications for the fate of the desert lakes.

## Results

### ICESat Data Study

The Ice, Cloud, and land Elevation Satellite (ICESat) mission was launched in January 2003 and ended in February 2010, providing a global altimeter data set for many applications with an unprecedented elevation accuracy of up to 2 cm^12^,^13^. About 30 ICESat satellite tracks cross the lake area in the BJD. Thirteen lakes intercept ICESat footprints, seven with footprints at only one snapshot and six with footprints from at least three different times. The water level data estimated from ICESat are presented in Supplementary Table S1.

**Figure 1:**
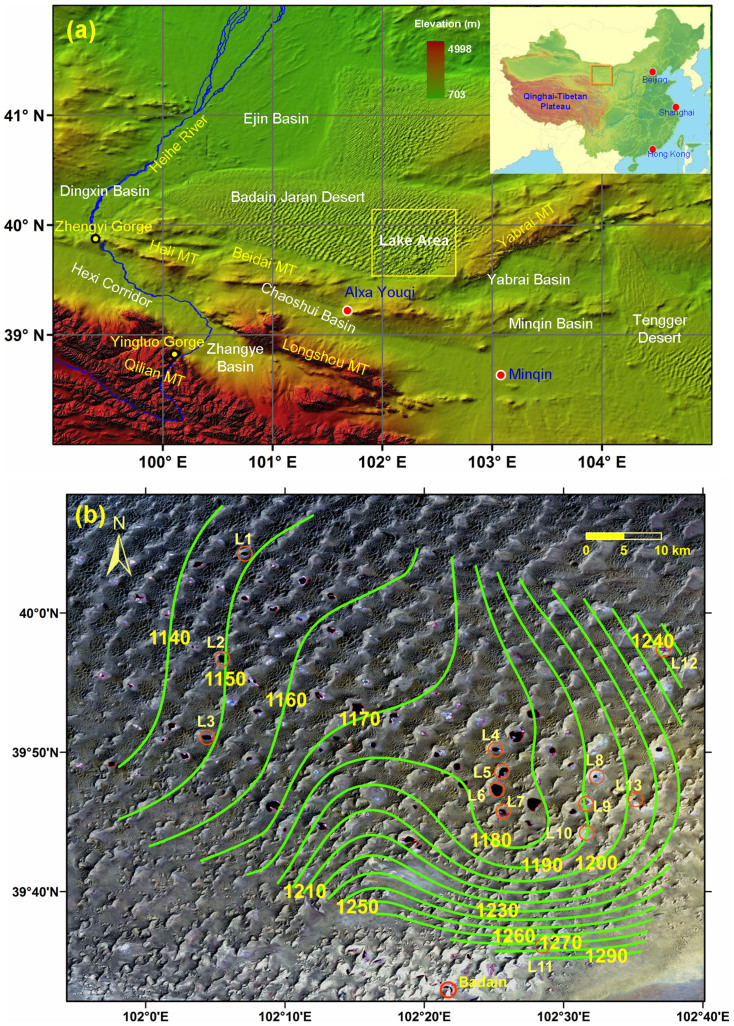
(a) The Badain Jaran Desert and the lake area; (b) Landsat Thematic Mapper image of the areas with most of the lakes in the desert on May 24, 2003, together with the groundwater contour map (water level interval = 10 m) based on the water level estimated from ICESat data. Spline interpolation method was used to interpolate the water level data to produce the map. Lakes that are discussed in detail are marked with circles (Both maps are generated in ArcGIS 9).

**Figure 2:**
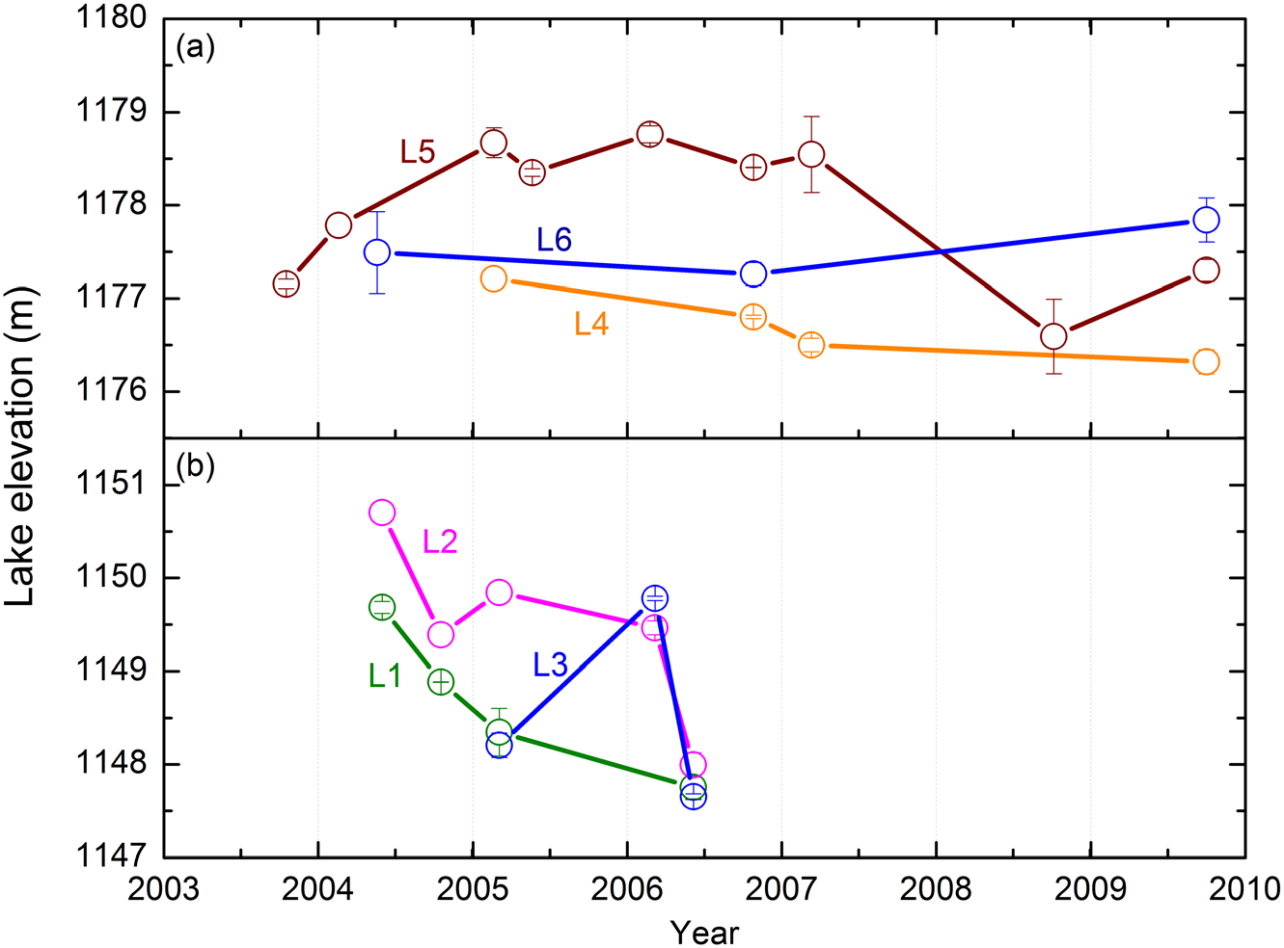
Temporal water level estimated from ICESat data for lakes L4 to L6 (a) and lakes L1 to L3 (b) from 2003 to 2009.

Temporal changes in water levels in lakes L1 to L6 are presented in Fig. 2. Lakes L1 to L3 are located in the northwest of the lake area and L4 to L6 in the southeast (Fig. 1b). Because water level changes in most of the lakes are much greater than the typical annual change of 25 cm^14^, these changes reflect the inter-annual variation of groundwater conditions in the relatively long time period.

**Figure 3:**
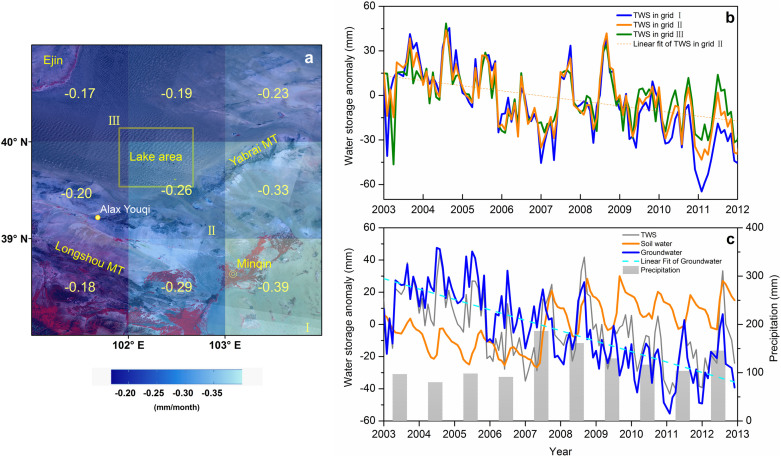
(a) Spatial distribution of changing TWS rate estimated from GRACE in regions around the lake area; (b) Temporal TWS change in grids I, II, and III; (c) Changes in TWS, groundwater storage, and soil water in grid I, together with temporal changes in precipitation.

Water level data are available for lakes L1 to L3 for the period from 2004 to 2006. During this period, water level in L1 and L2 decreases while the trend in L3 is unclear. L4 to L6 have water level data spanning a much longer period. The water level decreases in L4 but increases in L6. The water level in L5 is over 1178 m from 2005 to 2007 but thereafter drops by about 1 m despite the significant increase in the rainfall between 2007 and 2009 (Fig. 3b). On the basis of water level changes in Fig. 2, the overall water level in the lake area is decreasing. This conclusion is also supported by the shrinking of the lake areas from 2000 to 2010 based on remote sensing images of the lakes^15^.

Because the lakes are mainly fed by groundwater, water levels from different lakes should reflect the spatial distribution of the groundwater water level and the flow direction. While lake level data are obtained for different times, the temporal change in lake level is much less than the spatial change of the water level when the lakes are far from each other (Table S1). For example, the distance between the L1 to L3 group of lakes and the L4 to L6 group of lakes is about 32 km. The water levels of these two groups range from 1148.55 m to 1149.48 m and from 1176.71 m to 1177.95 m, respectively.

Fig. 1b presents the water level contour map based on the estimated lake water level. The general flow direction is from southeast to northwest. The contour lines around lakes L4–L7 indicate flow towards these lakes, possibly induced by evaporation from the lakes. A more permeable zone appears to exist between lakes L13 and L2, with the average hydraulic gradient between these two lakes at about 1/1000. The source of recharge is mainly from the mountains (e.g., Yabrai Mountains) to the south and the east.

### GRACE data study

The Gravity Recovery and Climate Experiment (GRACE) mission, launched by NASA and the German Aerospace Centre in May 2002, features twin co-orbiting satellites that in tandem measure the Earth's gravity field with unprecedented accuracy and can provide vertically integrated TWS change after adjusting for nonhydrologic effects^13^,^16^. Data representing a total of 115 monthly TWS anomalies since January 2003 were used in this study^17^.

The TWS variability mainly consists of components including variations of surface water, groundwater, soil moisture, and snow water equivalent^18^,^19^,^20^. As no surface runoff occurs in the desert and snow water is negligible, TWS estimated from GRACE is attributed to changes in soil water and groundwater. GRACE data in nine grids centered at the lake area in the BJD were analyzed. Fig. 3a shows the average changing rate of the monthly TWS from 2003 to 2012 over these grids, all of which are negative. The rate is progressively more negative moving from northwest to southeast. Grid I, which includes part of the Minqin Basin and Tengger Desert, has the decreasing TWS of the largest magnitude (−0.39 mm/month). The grids near the Qilian Mountains and the lower reach of the Heihe River have decreasing rates of smaller magnitude (around −0.20 mm/month).

The TWS changes in grids I, II, and III (Fig. 3b) respond closely on a monthly basis to precipitation (Fig. S1). A major decrease in TWS occurred around 2006 when the rainfall was low and a rebound occurred in 2007, 2008 and 2012 when rainfall was high. Fig. 3b shows the difference among the TWS in the three grids is small in the early years but becomes larger after 2009, suggesting the water depletion is worsening in grid I or/and is improving in grid III.

### Groundwater storage

Groundwater storage variations can be isolated from TWS if other components can be identified from either in situ observations or land-surface models. Soil moisture and snow water equivalent data can be obtained from the products of the Global Land Data Assimilation System (GLDAS). Because the temporal TWS changes in grids I to III are similar, groundwater changes in these grids are also expected to be similar. Figure 3c plots the 10-yr time series of groundwater, soil water, and TWS changes for grid II, which covers most of the lakes in the desert. Variations in groundwater, TWS, and rainfall correspond at the monthly scale. Both groundwater storage and TWS approach a maximum in July when rainfall is the greatest (Fig. S1), suggesting that changes in both groundwater storage and TWS are driven by rainfall or that a substantial portion of the rainfall has recharged the groundwater.

Being closely related to rainfall (Fig. S1), the soil water is low from 2003 to 2006 and then significantly increased after 2007 when rainfall increased. This suggests that soil water is entirely controlled by local climate. The increase in rainfall from 2007 to 2012 increased the groundwater storage, as seen from the large increase of groundwater storage in those wetter years (Fig. 3c); however, groundwater storage is decreasing over the long term. This suggests that, unlike soil water, groundwater storage is controlled by more than just the local climate. Groundwater pumping in the desert is negligible, so factors beyond the desert must be leading to a decrease in groundwater storage. The average rate of depletion of groundwater between 2003 and 2012 is estimated at 6.54 mm yr^−1^ equivalent height of water, or 29 Mm^3^yr^−1^ for the total lake area (yellow square in Fig. 3a) of about 4400 km^2^. This rate might have been larger if not for the overall increase in rainfall between 2007 and 2012. Assuming the mean porosity of the desert is 0.3, the average decline in groundwater level is about 21.8 mm/year. This slow but long-term reduction in groundwater storage is believed to be the cause of the decrease in water level and area of the lakes, especially the many small and shallow lakes.

## Discussion

According to the GRACE data, both TWS and groundwater storage are decreasing (Fig. 3b,c), but not all lakes show a decrease in water level (Fig. 2). The lakes may be partially recharged by local precipitation^21^ in the lake catchments and partially by deep confined aquifers, as indicated by the widely observed upward-flowing springs around and inside some lakes. These springs have recharge from deep confined aquifers with a water age of at least several decades^6^,^11^. Thus, lake levels do not show an immediate response to the water storage changes indicated by the GRACE data. The lakes that show an obvious water level decline could be mainly recharged from nearby sources, while those that do not show an immediate reduction could be recharged predominantly by deep confined aquifers with sources in the nearby mountainous areas.

Previous work shows that groundwater from neighboring basins, such as the Chaoshui Xi Basin, the Chaoshui Dong Basin, and the Yabrai Basin (Fig. 1a), might feed the lakes in the BJD^2^,^11^,^22^. The water level in the desert is very likely influenced by groundwater movement from areas to the southeast, especially the Yabrai Mountains (Fig. 1b).

Surface water inflow to the Minqin Basin has declined to about 100–150 million cubic meters per year (Mm^3^/year) in recent decades, indicating that groundwater is extensively pumped for agriculture and industry purposes^21^. Modeling shows that the aquifer has an average deficit of 260 Mm^3^/year^21^. Thus, significant groundwater depletion in these basins might cause a decrease in TWS and groundwater storage in the southeastern grids (Fig. 3a).

The Heihe River forms the key hydraulic boundary on the west and southwest of the desert. To restore the ecosystem in the Lower Heihe River (north to the Zhengyi Gorge; see Fig. 1a), water regulation has been in force since 2000; water consumption in the Middle Heihe River (between Zhengyi and Yingluo gorges) has been reduced and more water is delivered to the Lower Heihe River^23^. Groundwater is still pumped in the Dingxin Basin and the Ejina Oasis, however, and this causes a water balance deficit^23^.

To the southwest of the desert, the Zhangye Basin experienced a regional groundwater level decrease of 4 to 6 m from the 1980s to 2003 due to extensive groundwater abstraction^24^,^25^. However, the water level started to increase after 2003. Glacier melt from the Qilian Mountains and precipitation increase as a result of changing climate are believed to have augmented groundwater recharge and river runoff^26^. The effect of long-term groundwater abstraction in the west and southwest neighboring areas may be negated to some degree by the effect of extra water supply to these areas due to snow and ice melt from the Qilian Mountains in the past decade or so. This is reflected in the smaller TWS reduction in the grids neighboring these areas (Fig. 3a). The gradual decrease in groundwater storage revealed in this study does not support the hypothesis that the desert has received a large amount of recharge originating from the Qilian Mountains or Qinghai-Tibetan Plateau^9^,^27^ through deep fault systems.

We conclude that both local rainfall recharge and regional recharge from the southeast are significant. Short-term seasonal groundwater storage changes are controlled by local precipitation and multi-year variations in regional flow. If this decreasing trend in groundwater storage continues, the lakes will shrink or even eventually disappear, leading to adverse consequences such as the deterioration or even permanent devastation of the oasis ecology and environment.

## Methods

The ICESat mission provided elevation data over all Earth's surface in 19 successful global campaigns from 2003 to 2009. The ICESat/GLA14 Release-33 elevation data covering the study region for the period 2003–2009 were downloaded through the U.S. National Snow and Ice Data Center (NSIDC). Using NSIDC GLAS Altimetry Elevation Extractor Tool (NGAT), the elevation data, with latitude, longitude, geoid height and saturation corrections can be extracted. The GLAS elevations are referenced to the Topex/Poseidon ellipsoid and EGM96 Geoid. We converted them to the WGS84 ellipsoid elevations, which is a common coordinate frame and used in Landsat images and DEM.

Using Landsat TM/ETM+ imagery data of the same period with ICESat, we obtained the boundaries of the lakes. ICESat data of tracks intersecting with each lake were extracted. The extracted footprints within each lake were carefully examined to remove outliers. A two-step procedure^12^ was used for outlier removal. The water levels of each track were averaged and time-series data for each lake were constructed. We then analyzed the changes of water levels and obtained the contour map from multi-year averaged data.

A total of 115 monthly TWS anomalies of Release 05 of the latest version from NASA Jet Propulsion Laboratory (JPL) at California Institute of Technology was used in this study. The CSR monthly solutions are based on the CSR spherical harmonics and are filtered to minimize the effect of North-South stripes. The data are further corrected for glacial isostatic adjustment and smoothed with a Gaussian filter of 300 km radius^17^. Since the spatial resolution of GRACE is 1° of the latitude and longitude, the study area is also divided into cells with 1° cell size of about 100 km × 100 km. The filtering of GRACE data is typically done at 300 km, which is about 3° in the geographic coordinate system and the error of the TWS estimated from the GRACE data at this resolution is less than 1 cm of equivalent water height^28^.

TWS observation includes the combined contributions of groundwater, soil water, surface water, snow, ice and biomass. Groundwater storage variations can be isolated from TWS if other components can be identified from either in situ observations or land-surface models. In the desert area, the TWS variability was assumed to be mainly caused by the variations of groundwater, soil moisture, and snow water equivalent^18^,^19^. Thus, groundwater storage changes were estimated by subtracting soil moisture and snow water from TWS. Although the 1° × 1°resolution of GRACE-derived data are coarse, the time series data in and around the lake area assisted in analyzing the lake level changes.

The soil moisture and snow water equivalent data were from the products of the GLDAS (Global Land Data Assimilation System), which is on the global of 1° × 1°grids. GLDAS modelled soil-water fields integrate the effect of precipitation, solar radiation, air temperature and other meteorological factors^20^. The GLDAS Noah monthly data, spanning from January 2003 to December 2012, the same with GRACE data, were prepared as the anomalies with respect of the multi-year mean (mm).

## Supplementary Material

Supplementary Information Supplementary information
