# Supplementary material for: Satellite-based estimates of groundwater depletion in the Badain Jaran Desert, China
Source: Sci Rep. 2015 Mar 11;5:8960. doi: 10.1038/srep08960 (PMC5390913; doi:10.1038/srep08960)
Supplement: Supplementary Information [file srep08960-s1.doc]

Supplementary Information

# Satellite-based estimates of groundwater depletion in the Badain Jaran Desert, China

Jiu Jimmy Jiao^1*^, Xiaotao Zhang^1^, and Xusheng Wang^2^

^1^ Department of Earth Sciences, The University of Hong Kong, Hong Kong, China

^2^ School of Water Resources & Environment, China University of Geosciences, Beijing, China


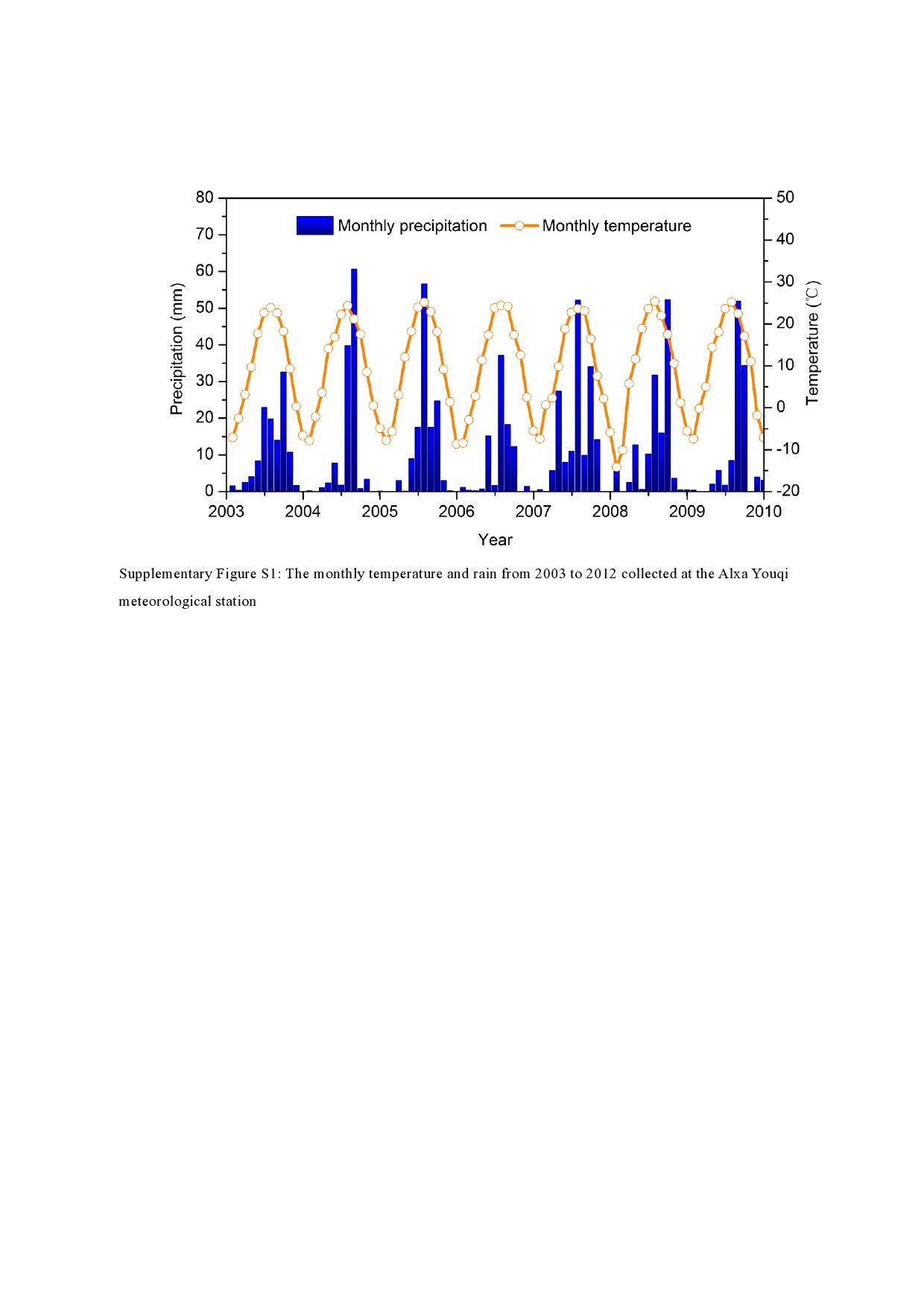


Supplementary Table S1: Lake water level derived from ICESat data and standard derivations of the estimated water level

| Lake ID  (local name) | Date | No. of footprints | Estimated  lake level (m) | STD of estimated water level (m) | Mean level (m) |
| --- | --- | --- | --- | --- | --- |
| Lake 1  (Baganaogunnuoer) | 5/31/2004 | 2 | 1149.68 | 0.06 | 1148.67 |
|  | 10/17/2004 | 2 | 1148.89 | 0.00 |  |
|  | 3/4/2005 | 2 | 1148.35 | 0.26 |  |
|  | 6/6/2006 | 2 | 1147.75 | 0.12 |  |
| Lake 2  (Nuoertu) | 5/31/2004 | 1 | 1150.70 | - | 1149.48 |
|  | 10/17/2004 | 1 | 1149.39 | - |  |
|  | 3/4/2005 | 1 | 1149.84 | - |  |
|  | 3/7/2006 | 4 | 1149.47 | 0.08 |  |
|  | 6/6/2006 | 4 | 1147.99 | 0.13 |  |
| Lake 3  (Shaoergetu) | 3/4/2005 | 2 | 1148.21 | 0.13 | 1148.55 |
|  | 3/7/2006 | 3 | 1149.78 | 0.02 |  |
|  | 6/6/2006 | 2 | 1147.65 | 0.03 |  |
| Lake 4  (Xiaohaizi) | 2/19/2005 | 1 | 1177.21 | - | 1176.71 |
|  | 10/26/2006 | 2 | 1176.80 | 0.02 |  |
|  | 3/12/2007 | 2 | 1176.50 | 0.07 |  |
|  | 10/1/2009 | 2 | 1176.32 | 0.13 |  |
| Lake 5  (Sumu Jaran) | 10/17/2003 | 2 | 1177.16 | 0.05 | 1177.95 |
|  | 2/18/2004 | 1 | 1177.78 | - |  |
|  | 2/19/2005 | 6 | 1178.67 | 0.16 |  |
|  | 5/21/2005 | 3 | 1178.35 | 0.04 |  |
|  | 2/23/2006 | 2 | 1178.76 | 0.09 |  |
|  | 10/26/2006 | 2 | 1178.41 | 0.00 |  |
|  | 3/12/2007 | 3 | 1178.55 | 0.41 |  |
|  | 10/5/2008 | 2 | 1176.59 | 0.40 |  |
|  | 10/1/2009 | 2 | 1177.30 | 0.12 |  |
| Lake 6  (Barun Jaran) | 5/19/2004 | 5 | 1177.49 | 0.44 | 1177.53 |
|  | 10/26/2006 | 2 | 1177.26 | 0.13 |  |
|  | 10/1/2009 | 2 | 1177.84 | 0.24 |  |
| Lake 7  (Maoritu) | 5/19/2004 | 2 | 1175.27 | 0.12 | 1175.27 |
| Lake 8  (Hulongte) | 3/11/2003 | 2 | 1192.61 | 0.32 | 1192.61 |
| Lake 9 | 9/27/2003 | 1 | 1190.43 | - | 1190.43 |
| Lake 10 | 2/23/2003 | 1 | 1189.86 | - | 1189.86 |
| Lake 11  (Haisen Jaran) | 10/22/2005 | 1 | 1274.02 | - | 1274.02 |
| Lake 12 | 10/5/2003 | 2 | 1242.68 | 0.11 | 1242.68 |
| Lake 13  (Zhamuhuduge) | 10/5/2003 | 1 | 1202.54 | - | 1202.54 |

(Note: when there is more than one footprint used in estimating the water level, the mean standard deviation (STD) of the estimates is calculated. The symbol “-” means data not available)
